# Medium-term clinical and imaging outcomes of SilverFlow covered bridging stent implantation using *in situ* fenestration for aortic arch lesions: a single-centre retrospective cohort study

**DOI:** 10.3389/fcvm.2026.1945990

**Published:** 2026-08-31

**Authors:** Xiangyang Shu, Daping Zhang, Pengfei Zhou, Zongjiang Yang, Zhaoxiong Deng, Guangning Liu

**Affiliations:** Department of Vascular Surgery, People’s Hospital of Qiandongnan Miao and Dong Autonomous, Prefecture, Kaili, Guizhou, China

**Keywords:** aortic arch, aortic remodelling, covered bridging stent, *in situ* fenestration, thoracic endovascular aortic repair

## Abstract

**Introduction:**

Evidence regarding the medium-term performance of SilverFlow covered bridging stents for in-situ fenestrated aortic arch repair remains limited. This study evaluated the clinical and imaging outcomes of SilverFlow covered bridging-stent implantation for the treatment of aortic arch lesions.

**Methods:**

This single-centre, single-arm retrospective cohort study included consecutive adult patients who underwent in-situ fenestrated aortic arch repair with SilverFlow covered bridging stents between January 2023 and April 2025. The primary outcome was technical success. Secondary outcomes included 30-day major adverse events, mortality, target-vessel patency, aortic remodelling, and reintervention rates.

**Results:**

Eighty-one patients received 140 SilverFlow covered bridging stents. Technical success was achieved in 70 patients (86.4%). Successful stent deployment, completion of planned fenestrations, and immediate target-vessel patency were each achieved in 98.8% of cases. No intraoperative, in-hospital, or 30-day deaths occurred. The 30-day major adverse-event rate was 25.9%. At 12 months, all 107 assessed branch vessels remained patent. At the latest imaging follow-up, per-vessel and patient-level patency rates were 98.5% and 97.5%, respectively. Significant reductions were observed in maximum aortic diameter (−4.0 mm, P = 0.020), false-lumen diameter (−6.8 mm, P < 0.001), and aneurysm-sac diameter (−8.5 mm, P = 0.010). Complete false-lumen thrombosis increased from 32.5% to 55.0% (P = 0.020). During a median follow-up of 19.0 months, four patients died and seven required aortic- or branch-related reintervention. Freedom from reintervention was 92.4% at 12 months and 90.8% at 24 months.

**Discussion:**

In this single-centre cohort, in-situ fenestrated aortic arch repair using SilverFlow covered bridging stents demonstrated high immediate reconstruction success, durable target-vessel patency among assessed vessels, and favourable imaging indicators of aortic remodelling during medium-term follow-up. These findings support the feasibility of this technique; however, comparative studies are required to determine its relative effectiveness. Continued imaging surveillance remains essential because reinterventions and device- or aorta-related abnormalities occurred during follow-up.

## Introduction

Aortic arch aneurysms and dissections are among the most challenging aortic conditions because treatment must exclude the diseased segment while preserving cerebral, spinal-cord, and upper-extremity perfusion through the supra-aortic branches (1–3). The complex curvature of the arch, variable branch-vessel anatomy, and substantial haemodynamic forces further complicate both open and endovascular reconstruction (2–4). In aortic dissection, treatment must also exclude the primary entry tear, restore true-lumen flow, reduce false-lumen pressurisation, and promote favourable aortic remodelling (1, 2).

Open total arch replacement remains an established treatment for extensive aortic arch disease but generally requires sternotomy, cardiopulmonary bypass, cerebral-protection strategies, and hypothermic circulatory arrest (1–3). Despite improvements in surgical techniques, open repair remains associated with risks of mortality, neurological injury, renal dysfunction, respiratory complications, bleeding, and prolonged recovery, particularly in older patients and those with substantial comorbidities (1–3). Hybrid and total endovascular approaches have therefore been developed as less-invasive alternatives for appropriately selected patients (2–4).

The use of thoracic endovascular aortic repair in the aortic arch is limited by the need for an adequate proximal sealing zone, which may require intentional coverage of one or more supra-aortic branch vessels (3–5). Preservation of these vessels can be achieved using surgical debranching, parallel grafts, physician-modified devices, custom-manufactured fenestrated or branched endografts, or *in-situ* fenestration (3–5). Custom-manufactured devices permit patient-specific reconstruction but are limited by production time and may not be suitable for urgent or emergency presentations (3, 4). Parallel-graft and physician-modified techniques are more readily available but may be associated with gutter-related endoleaks, difficulties in device orientation, and variability in procedural performance (3, 4).

In-situ fenestration involves deployment of the Ankura thoracic stent graft across the target-vessel origin, followed by retrograde penetration of the graft fabric and implantation of a covered bridging stent to restore target-vessel perfusion (5–8). Fenestration can be performed using a mechanical needle, laser catheter, radiofrequency device, or another dedicated puncture system and may be used for single- or multivessel supra-aortic reconstruction (5, 6). Because the fenestration is created after deployment of the main endograft, the reconstruction can be adapted to the patient's intraoperative anatomy using readily available devices (5, 6). These features make *in-situ* fenestration a potentially useful option for complex or urgent aortic arch lesions when treatment cannot be delayed for the manufacture of a customised device (6–9).

Nevertheless, *in-situ* fenestration is technically demanding and may be complicated by cerebral embolisation, temporary interruption of branch perfusion, inaccurate graft penetration, bridging-stent compression or kinking, endoleak, migration, stenosis, occlusion, and subsequent reintervention (5–9). Previous observational studies and systematic reviews have generally reported technical success rates exceeding 90% and favourable short- to medium-term target-vessel patency (6–10). However, the available evidence is derived predominantly from retrospective single-centre studies with heterogeneous aortic pathologies, fenestration methods, devices, outcome definitions, and follow-up durations (5, 6, 8, 9). Evidence regarding medium-term bridging-stent durability, aortic remodelling, false-lumen thrombosis, and freedom from reintervention therefore remains limited (8, 9).

The SilverFlow covered stent has been used as a bridging stent to preserve supra-aortic target-vessel perfusion following *in-situ* fenestration, but device-specific clinical evidence remains limited (11). Few studies have comprehensively evaluated procedural outcomes, early safety, longitudinal target-vessel patency, aortic remodelling, haemodynamic changes, and reintervention within the same cohort (8, 9, 11). Therefore, this single-centre retrospective cohort study aimed to evaluate the medium-term clinical and imaging outcomes of SilverFlow covered bridging-stent implantation using *in-situ* fenestration for aortic arch lesions, focusing on technical success, early adverse events, target-vessel patency, aortic remodelling, mortality, and freedom from aortic- or branch-related reintervention.

## Methods

### Study design and setting

This single-centre retrospective cohort study included consecutive adult patients who underwent SilverFlow bridging-stent implantation using an *in-situ* fenestration technique for aortic arch lesions at People's Hospital of Qiandongnan Miao and Dong Autonomous Prefecture, Kaili, Guizhou, China. between 1 January 2023 and 15 April 2025. This was a single-arm observational cohort; no concurrent open, hybrid, chimney, or alternative endovascular comparator group was available. The administrative follow-up cutoff was 15 April 2026, providing at least 12 months of potential follow-up for all included patients. The study was conducted in accordance with the Declaration of Helsinki and was approved by the Institutional Review Board of Qiandongnan Miao and Dong Autonomous Prefecture People's Hospital (approval no. 2021-12-003). The requirement for informed consent was waived due to the retrospective study design.

No formal sample-size calculation was performed because all eligible patients treated during the prespecified study period were included.

### Study population and patient characteristics

Patients were eligible if they were aged 18 years or older, had an aortic arch dissection or aneurysm requiring endovascular treatment, underwent implantation of one or more covered SilverFlow bridging stents using an *in-situ* fenestration technique, completed the index procedure during the study inclusion period, and had sufficient operative and clinical records to determine procedural and in-hospital outcomes.

Patients who did not undergo the planned procedure were not included in the analysis. Reasons for exclusion included anatomy unsuitable for *in-situ* fenestration, an insufficient landing zone, active infection, contraindication to contrast-enhanced imaging, connective-tissue disorders, preference for open surgery, death before the planned intervention, or refusal of the procedure.

A total of 105 patients were screened for eligibility. Of these, 24 were excluded and 81 underwent the index SilverFlow procedure and were included in the final analysis.

### Data collection and quality control

Patient-level data were retrospectively extracted from the institutional screening log, electronic medical records, operative and anaesthetic records, fluoroscopy logs, implant records, intensive care and discharge records, outpatient follow-up notes, and computed tomography angiography archives. Each patient was assigned a deidentified study code.

Baseline data included age, sex, body mass index, presenting symptoms, procedural indication, lesion type, dissection chronicity, aneurysm morphology, previous aortic intervention, smoking status, comorbidities, blood-pressure measurements, antihypertensive treatment, echocardiographic measurements, and baseline aortic anatomy.

Procedural data included procedure urgency, anaesthesia type, vascular access, lesion and landing-zone locations, Ishimaru zone, aortic dimensions, fenestration method, target vessels, number of fenestrations, bridging-stent dimensions, systemic heparin administration, maximum activated clotting time, balloon dilatation, residual pressure gradient, procedure duration, fluoroscopy time, contrast volume, estimated blood loss, completion angiographic findings, and technical success.

Clinical events, complications, deaths, and reinterventions were identified from all available source documents and linked using the patient study code. Before final data lock, internal consistency was assessed across the patient, procedural, device, follow-up, imaging, complication, and reintervention datasets. Any unresolved discrepancies were checked against the original source records.

### Device selection and procedural planning

Preoperative computed tomography angiography was used to evaluate aortic arch morphology, proximal and distal landing-zone dimensions, target-vessel diameters, and the relationship of the supra-aortic vessels to the intended sealing zone. The Ankura Thoracic Stent Graft System (LifeTech Scientific, Shenzhen, China) was used as the main aortic endograft. The Ankura device is a self-expanding thoracic stent graft comprising a nitinol framework with an ePTFE covering. A proximal sealing length of approximately 15 mm was generally required when anatomically feasible. Ankura endograft diameter was selected according to landing-zone measurements and underlying pathology, with approximately 10% oversizing for aortic dissection and approximately 20% for aneurysmal disease (12). Final graft selection was individualized according to aortic morphology and operator judgement.

SilverFlow covered stents (LifeTech Scientific, Shenzhen, China) were used as bridging covered stents between the *in-situ* fenestration and the reconstructed supra-aortic target vessel. SilverFlow is a self-expanding covered stent based on a nitinol framework with an ePTFE covering. SilverFlow Bridging-stent diameter was selected according to the measured target-vessel diameter and was generally equal to or approximately 1–2 mm larger than the target vessel. Stent length was selected to provide secure distal fixation within the native target vessel while maintaining approximately 10 mm of proximal extension across the fenestration into the Ankura thoracic stent graft/aortic lumen (13). Care was taken to avoid compromising adjacent branch origins. In this cohort, the median nominal SilverFlow diameter was 10.0 mm [IQR 8.0–12.0] and the median nominal length was 50.0 mm [40.0–60.0].

### Endovascular procedure

All procedures were performed by an experienced multidisciplinary aortic team under general anaesthesia, sedation, or local anaesthesia according to the patient's clinical condition and operator judgement. Vascular access was obtained through percutaneous or surgical femoral access, with additional iliac, brachial, or axillary access when required.

The Ankura thoracic endograft was advanced through the femoral access and deployed across the diseased aortic arch segment, with intentional coverage of the supra-aortic target-vessel origin(s) when required to obtain an adequate proximal sealing zone. After deployment, the target vessel was accessed retrogradely through the established branch-vessel access and the endograft fabric was approached as perpendicularly as anatomy permitted. *In-situ* fenestration was performed using either a laser-assisted or adjustable needle-puncture technique according to the patient's anatomy and operator judgement. In laser-assisted procedures, the graft fabric was penetrated using short pulses of laser energy at approximately 18 W. In needle procedures, an adjustable puncture system was positioned against the graft fabric before controlled mechanical penetration. After successful penetration and guidewire passage into the aortic lumen, the initial fenestration was enlarged with a small balloon, generally beginning at approximately 4 mm, followed by progressive balloon dilatation according to the target-vessel diameter (13). A SilverFlow covered bridging stent was then advanced through the fenestration and deployed between the Ankura endograft and the native target vessel. The stent was positioned to maintain secure distal fixation within the native vessel and approximately 10 mm of proximal extension across the fenestration into the aortic endograft/aortic lumen. Reconstruction was performed for the innominate artery, left common carotid artery, and/or left subclavian artery according to the planned repair.

Systemic heparin was administered unless clinically contraindicated, and activated clotting time was monitored intraoperatively. After SilverFlow deployment, bridging-stent post-dilatation was performed selectively when angiography demonstrated incomplete expansion, residual stenosis, inadequate fenestration enlargement, or suboptimal apposition (13). Balloon diameter was selected according to the reconstructed target-vessel and bridging-stent diameter, with care to avoid excessive oversizing. Additional balloon dilatation or stent optimization was performed when impaired target-vessel flow or persistent structural compromise remained. Completion angiography was performed to confirm main-endograft position, completion of all planned fenestrations, unrestricted target-vessel flow, satisfactory bridging-stent expansion and apposition, and the absence of clinically significant residual stenosis, major type I or III endoleak, or vascular injury. Additional balloon dilatation or endovascular correction was performed when required. Postprocedural antiplatelet therapy was administered according to the institutional protocol and the individual patient's bleeding risk.

### Follow-up

The institutional follow-up schedule included assessment at discharge or approximately 30 days, at approximately 6 months, at 12 months, and annually thereafter. Actual assessment dates were retained in the analytical dataset.

The prespecified assessment windows were 4–8 months for the 6-month visit, 10–14 months for the 12-month visit, 21–27 months for the 24-month visit, and 33–39 months for the 36-month visit. Only assessments occurring within the 10–14-month window were included in the formal baseline-to-12-month paired analyses. An earlier or later assessment was not substituted when no assessment was available within the prespecified 12-month window.

The latest follow-up was defined separately for clinical, echocardiographic, aortic-imaging, and target-vessel outcomes as the latest valid dated assessment available on or before 15 April 2026.

Clinical follow-up included vital status, blood pressure, hypertension status, antihypertensive medication use and medication count, neurological symptoms, upper-limb ischaemia, readmission, and subsequent intervention. Imaging surveillance was primarily performed using computed tomography angiography.

### Imaging assessment

Aortic imaging was evaluated at baseline and during follow-up for maximum aortic diameter, true-lumen diameter, false-lumen diameter, aneurysm-sac diameter, false-lumen status, exclusion of the primary entry tear, endoleak, aortic remodelling, pseudoaneurysm formation, and main-graft migration or fracture.

False-lumen status was classified as patent, partially thrombosed, or completely thrombosed. Endoleaks were classified as type I, type II, type III, or other. Continuous changes in maximum aortic diameter, true-lumen diameter, false-lumen diameter, and aneurysm-sac diameter were assessed using paired baseline and 12-month measurements.

Target-vessel and bridging-stent imaging was evaluated separately for each reconstructed vessel. Outcomes included target-vessel patency, percentage stenosis, occlusion, bridging-stent-associated endoleak, migration, fracture, and kinking or compression. Significant target-vessel stenosis was defined as a luminal reduction of at least 50%. Patency was reported both per assessed target vessel and per patient. Patients or target vessels without evaluable imaging within the specified follow-up window were not included in the corresponding imaging denominator. For the patient-level analysis, all assessed target vessels were required to be patent for the patient to be classified as having complete target-vessel patency.

Imaging examinations were reviewed by two reviewers along with the institutional imaging team, and disagreements were resolved by consensus. A statement regarding independent or blinded review should be included only if this process was used in the actual study.

### Study outcomes

The primary outcome was technical success, defined as successful deployment of the Ankura thoracic stent graft and all planned SilverFlow bridging stents, completion of all intended *in-situ* fenestrations, patency of all reconstructed target vessels on completion angiography, and absence of type I or III endoleak, intraoperative open conversion, or intraoperative death. Failure of any component, including a minor type Ia endoleak, was classified as technical failure. The principal secondary safety outcome was a 30-day major adverse-event composite comprising all-cause death, stroke or transient ischaemic attack, spinal-cord ischaemia, acute kidney injury, myocardial infarction, respiratory failure, retrograde type A dissection, major aortic injury, target-vessel occlusion, open conversion, or unplanned reintervention. Access-site complications were evaluated and reported separately and were not included in the prespecified 30-day major adverse-event composite. Other secondary outcomes included mortality, procedural and device-related complications, target-vessel patency and structural abnormalities, aortic endoleak and remodelling, changes in aortic and false-lumen dimensions, false-lumen thrombosis, haemodynamic and antihypertensive outcomes, echocardiographic changes, and freedom from first aortic- or branch-related reintervention. A qualifying reintervention was any endovascular or open procedure performed after the index operation to treat an aortic or branch-related complication. Only the first qualifying reintervention was included in the time-to-event analysis.

### Adverse-event classification and review

Complications were classified by severity according to the CIRSE Classification System (14). Events corresponding to CIRSE Grade ≥3 were classified as major complications; these included events requiring additional therapeutic intervention or hospitalization lasting more than 48 h and events resulting in persistent sequelae, severe disability, or death. CIRSE Grade 1–2 events were classified as minor complications.

Serious adverse events were classified according to ISO 14155 criteria (15) and included adverse events resulting in death; a life-threatening illness or injury; permanent or significant impairment of a body structure or function; unplanned inpatient hospitalization or prolongation of hospitalization; or medical or surgical intervention required to prevent a life-threatening condition or permanent impairment. Planned hospitalization associated with the index procedure alone was not considered a serious adverse event.

Device-related events were those considered probably or definitely attributable to the implanted endovascular device, including the Ankura thoracic stent graft or SilverFlow covered bridging stent, its implantation, or its performance. Procedure-related events were those considered probably or definitely attributable to the index endovascular procedure or an associated procedural step, including vascular access, guidewire or catheter manipulation, *in-situ* fenestration, balloon dilatation, stent deployment, contrast administration, or other perioperative procedural factors. Severity, seriousness, device relatedness, and procedure relatedness were evaluated separately and were therefore not mutually exclusive classifications. Potential complication events were reviewed by two study investigators according to these prespecified definitions, with disagreements resolved by consensus.

### Statistical analysis

Continuous variables were summarised as mean ± standard deviation or median with interquartile range, as appropriate, and categorical variables as frequencies and percentages. Variable-specific denominators were reported for incomplete data. Binary outcomes were presented with 95% Wilson confidence intervals.

Paired baseline and 12-month continuous outcomes were compared using two-sided Wilcoxon signed-rank tests. Only patients with measurements at baseline and within the prespecified 10–14-month window were included. Paired binary outcomes were analysed using the exact McNemar test, and changes in false-lumen status using the Stuart–Maxwell test. Latest-follow-up measurements were summarised descriptively because assessment times varied.

Exploratory subgroup analyses were performed to examine whether procedural complexity and early outcomes varied according to underlying pathology, dissection chronicity, or *in-situ* fenestration technique. The subgroup outcomes were procedure duration, technical success, and the 30-day major adverse-event composite. Underlying pathology was classified as aortic arch dissection, aortic arch aneurysm, or dissection with aneurysmal degeneration. Dissection chronicity was evaluated among patients with a dissection component as acute, subacute, or chronic. Procedure duration was compared across these three-category subgroups using the Kruskal–Wallis test, while technical success and 30-day major adverse events were compared using Fisher–Freeman–Halton exact tests. For fenestration technique, inferential comparison was restricted to laser versus needle fenestration using the Mann–Whitney U test for procedure duration and Fisher's exact test for categorical outcomes. The six procedures classified as ‘other’ were reported descriptively because this small category comprised heterogeneous techniques.

Freedom from first aortic- or branch-related reintervention was estimated using the Kaplan–Meier method. Patients without reintervention were censored at death, last clinical contact, or 15 April 2026, whichever occurred first. Estimates were reported at 12, 24, and 36 months with 95% confidence intervals and numbers at risk; the 36-month estimate was interpreted cautiously because only one patient remained at risk.

Missing data were analysed using available cases without imputation. No multiplicity adjustment was applied because secondary analyses were exploratory. All tests were two-sided, with *P* < 0.05 considered statistically significant. Multivariable modelling was not performed because only seven reintervention events occurred. Analyses were conducted in Python 3.10 using pandas, SciPy, statsmodels, and lifelines.

## Results

### Study population

**Figure 1:**
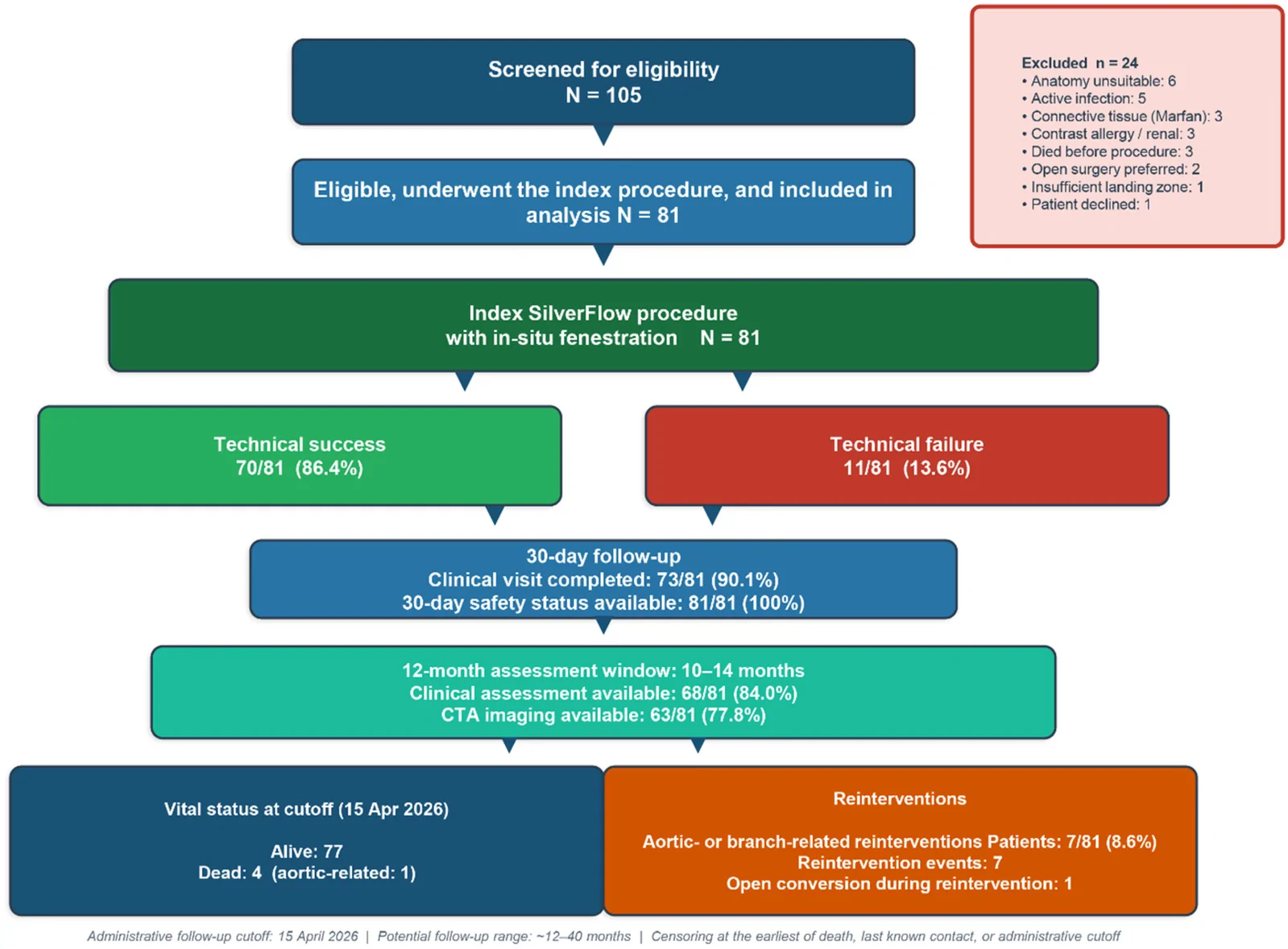
Study flow diagram.

A total of 105 patients were screened for eligibility. Twenty-four patients were excluded because of anatomy unsuitable for *in-situ* fenestration, active infection, connective-tissue disease, contrast allergy or renal contraindication, death before the planned procedure, preference for open surgery, an insufficient proximal landing zone, or refusal of treatment. The remaining 81 patients underwent the index SilverFlow procedure and were included in the analysis. Technical success was achieved in 70 patients, whereas 11 procedures met the prespecified definition of technical failure. Complete 30-day safety status was available for all patients, although an in-person 30-day clinical assessment was completed in 73 patients. Within the strict 10–14-month assessment window, clinical evaluation was available for 68 patients and computed tomography angiography was available for 63 patients. At the administrative cutoff of 15 April 2026, 77 patients were alive and four had died, including one aortic-related death. Seven patients underwent an aortic- or target-vessel-related reintervention, including one open conversion during reintervention (Figure 1).

### Baseline characteristics

The mean age of the study population was 64.5 ± 10.8 years, 19 patients (23.5%) were women, and the mean body mass index was 26.0 ± 4.7 kg/m^2^. The principal pathological categories were aortic arch dissection in 45 patients (55.6%), aortic arch aneurysm in 28 (34.6%), and dissection with aneurysmal degeneration in eight (9.9%). Fifty-seven patients (70.4%) were symptomatic at presentation, and 11 (13.6%) had undergone a previous aortic intervention.

Among the 53 patients with a dissection component, 15 (28.3%) had acute, 16 (30.2%) had subacute, and 22 (41.5%) had chronic dissection. Among the 28 patients with aneurysmal disease, 11 (39.3%) had saccular aneurysms, five (17.9%) had fusiform aneurysms, two (7.1%) had pseudoaneurysms, and 10 (35.7%) had other aneurysmal morphologies.

Hypertension was present in 64 patients (79.0%), diabetes mellitus in 18 (22.2%), coronary artery disease in 18 (22.2%), chronic kidney disease in 13 (16.0%), and previous stroke or transient ischaemic attack in 15 (18.5%). At baseline, 70 patients (86.4%) were receiving antihypertensive therapy, with a mean of 2.3 ± 1.3 medications. Mean upper- and lower-limb systolic blood pressures were 158.4 ± 16.6 and 146.3 ± 21.7 mmHg, respectively, corresponding to a mean upper-to-lower-limb systolic pressure gradient of 12.1 ± 12.6 mmHg. Baseline demographic, clinical, haemodynamic, and anatomical characteristics are presented in Table 1.

**Table 1:** Baseline characteristics.

Characteristic	Value	Available *n*
Demographics		
Patients	81	81
Age, years	64.5 ± 10.8	81
Female sex	19/81 (23.5%)	81
Body mass index, kg/m^2^	26.0 ± 4.7	81
Aortic pathology and presentation		
Aortic arch dissection	45/81 (55.6%)	81
Aortic arch aneurysm	28/81 (34.6%)	81
Dissection with aneurysmal degeneration	8/81 (9.9%)	81
Symptomatic at presentation	57/81 (70.4%)	81
Previous aortic intervention	11/81 (13.6%)	81
Dissection chronicity: acute	15/53 (28.3%)	53
Dissection chronicity: subacute	16/53 (30.2%)	53
Dissection chronicity: chronic	22/53 (41.5%)	53
Aneurysm morphology: fusiform	5/28 (17.9%)	28
Aneurysm morphology: saccular	11/28 (39.3%)	28
Aneurysm morphology: pseudoaneurysm	2/28 (7.1%)	28
Aneurysm morphology: other	10/28 (35.7%)	28
Comorbidities and smoking		
Hypertension	64/81 (79.0%)	81
Diabetes mellitus	18/81 (22.2%)	81
Coronary artery disease	18/81 (22.2%)	81
Chronic kidney disease	13/81 (16.0%)	81
Previous stroke or TIA	15/81 (18.5%)	81
Smoking: current	27/81 (33.3%)	81
Smoking: former	21/81 (25.9%)	81
Smoking: never	33/81 (40.7%)	81
Baseline clinical and hemodynamic characteristics		
Antihypertensive therapy	70/81 (86.4%)	81
Number of antihypertensive medications	2.3 ± 1.3	81
Upper-limb systolic BP, mmHg	158.4 ± 16.6	81
Lower-limb systolic BP, mmHg	146.3 ± 21.7	81
Upper-to-lower limb systolic BP gradient, mmHg	12.1 ± 12.6	81
Aortic velocity, m/s	2.3 ± 0.7	35
Peak aortic gradient, mmHg	23.2 ± 14.6	35
Baseline aortic anatomy		
Ascending aorta diameter, mm	34.7 ± 3.9	81
Proximal arch diameter, mm	36.5 ± 4.3	81
Distal arch diameter, mm	33.9 ± 3.6	81
Minimum aortic diameter, mm	26.9 ± 3.1	81
Descending aorta diameter, mm	30.7 ± 3.6	81
Proximal landing-zone length, mm	25.8 ± 5.5	81
Distal landing-zone length, mm	34.1 ± 8.5	81
Values are mean ± SD or *n*/*N* (%) unless otherwise indicated.		

### Procedural and technical outcomes

Procedures were performed electively in 43 patients (53.1%), urgently in 30 (37.0%), and as emergencies in eight (9.9%). General anaesthesia was used in 75 patients (92.6%). Femoral access was obtained percutaneously in 55 patients (67.9%) and through surgical cutdown in nine (11.1%), with iliac, brachial, or axillary access used in the remaining patients.

A single target vessel was treated in 38 patients (46.9%), two target vessels in 27 (33.3%), and three target vessels in 16 (19.8%). *In-situ* fenestration was performed using a laser in 47 patients (58.0%), a needle in 28 (34.6%), and another technique in six (7.4%). A total of 140 SilverFlow covered bridging stents were implanted, with a median of 2 bridging stents per patient. The reconstructed target-vessels comprised 50 innominate arteries, 50 left common carotid arteries, and 40 left subclavian arteries.

The median procedure duration was 203 min, median fluoroscopy time was 68.2 min, median contrast volume was 176 mL, and median estimated blood loss was 335 mL. Technical success was achieved in 70 of 81 patients (86.4%; 95% v CI 77.3–92.2). Deployment at the intended location, completion of all planned fenestrations, and patency of all target branches on completion angiography were each achieved in 80 of 81 patients (98.8%). Completion endoleak of any type was observed in 12 patients (14.8%), including seven minor type Ia endoleaks, three type III endoleaks, one type II endoleak, and one additional type I endoleak. There were no intraoperative deaths or open conversions during the index procedure. Detailed procedural, device, and technical outcomes are provided in Supplementary Table 1.

### Early safety outcomes and complications

The median length of hospital stay was 16 days, and 55 patients (67.9%) required admission to the intensive care unit. No in-hospital or 30-day deaths occurred. Eleven patients (13.6%) were readmitted within 30 days.

The 30-day major adverse-event composite occurred in 21 patients (25.9%; 95% CI, 17.6–36.4). Among the prespecified composite components, acute kidney injury occurred in nine patients (11.1%), respiratory failure in four (4.9%), stroke or transient ischaemic attack in three (3.7%), spinal-cord ischaemia in two (2.5%), aortic injury in two (2.5%), and target-vessel occlusion in two (2.5%). One patient required an unplanned reintervention within 30 days. No myocardial infarctions, retrograde type A dissections, open conversions, or deaths occurred. Access-site complications, which were reported separately and were not included in the composite, occurred in six patients (7.4%). Both target-vessel occlusions were managed conservatively because collateral perfusion was adequate and neither patient developed clinically significant cerebral or upper-limb ischemia. Neither occlusion required an additional endovascular or surgical procedure. One patient required an unplanned reintervention within 30 days. No myocardial infarctions or retrograde type A dissections were recorded.

Across the complete follow-up period, 24 patients (29.6%; 95% CI, 20.8–40.3) experienced at least one complication, accounting for 28 complication events. According to the prespecified severity, seriousness, and causality definitions, major complications occurred in 16 patients (19.8%), serious adverse events in 14 (17.3%), device-related events in six (7.4%), and procedure-related events in 20 (24.7%). These categories were not mutually exclusive. Safety, mortality, complication, and reintervention outcomes are detailed in Supplementary Table 2.

### Clinical and haemodynamic outcomes

Clinical assessment within the prespecified 12-month window was available for 68 patients. The median timing of this assessment was 11.7 months after the index procedure. In the paired cohort, mean upper-limb systolic blood pressure decreased from 159.0 ± 17.3 mmHg at baseline to 137.6 ± 13.4 mmHg at 12 months, with a paired mean change of −21.4 ± 21.0 mmHg (*P* < 0.001). Mean lower-limb systolic blood pressure decreased from 146.4 ± 22.9 to 128.3 ± 16.5 mmHg, corresponding to a paired change of −18.1 ± 26.3 mmHg (*P* < 0.001).

The upper-to-lower-limb systolic blood-pressure gradient decreased from 12.5 ± 13.2 to 9.3 ± 9.0 mmHg. However, the paired change of −3.2 ± 16.1 mmHg was not statistically significant (*P* = 0.100). The mean number of antihypertensive medications decreased from 2.3 ± 1.3 at baseline to 1.3 ± 1.2 at 12 months, with a paired change of −1.0 ± 1.9 medications (*P* < 0.001).

Among the 68 patients with paired hypertension data, the prevalence of hypertension decreased from 55 patients (80.9%) at baseline to 29 (42.6%) at 12 months (*P* < 0.001). Thirty-three patients changed from hypertensive to non-hypertensive status, whereas seven changed from non-hypertensive to hypertensive status. The proportion receiving antihypertensive therapy decreased from 60 of 68 patients (88.2%) to 43 of 68 (63.2%; *P* = 0.002).

**Figure 2:**
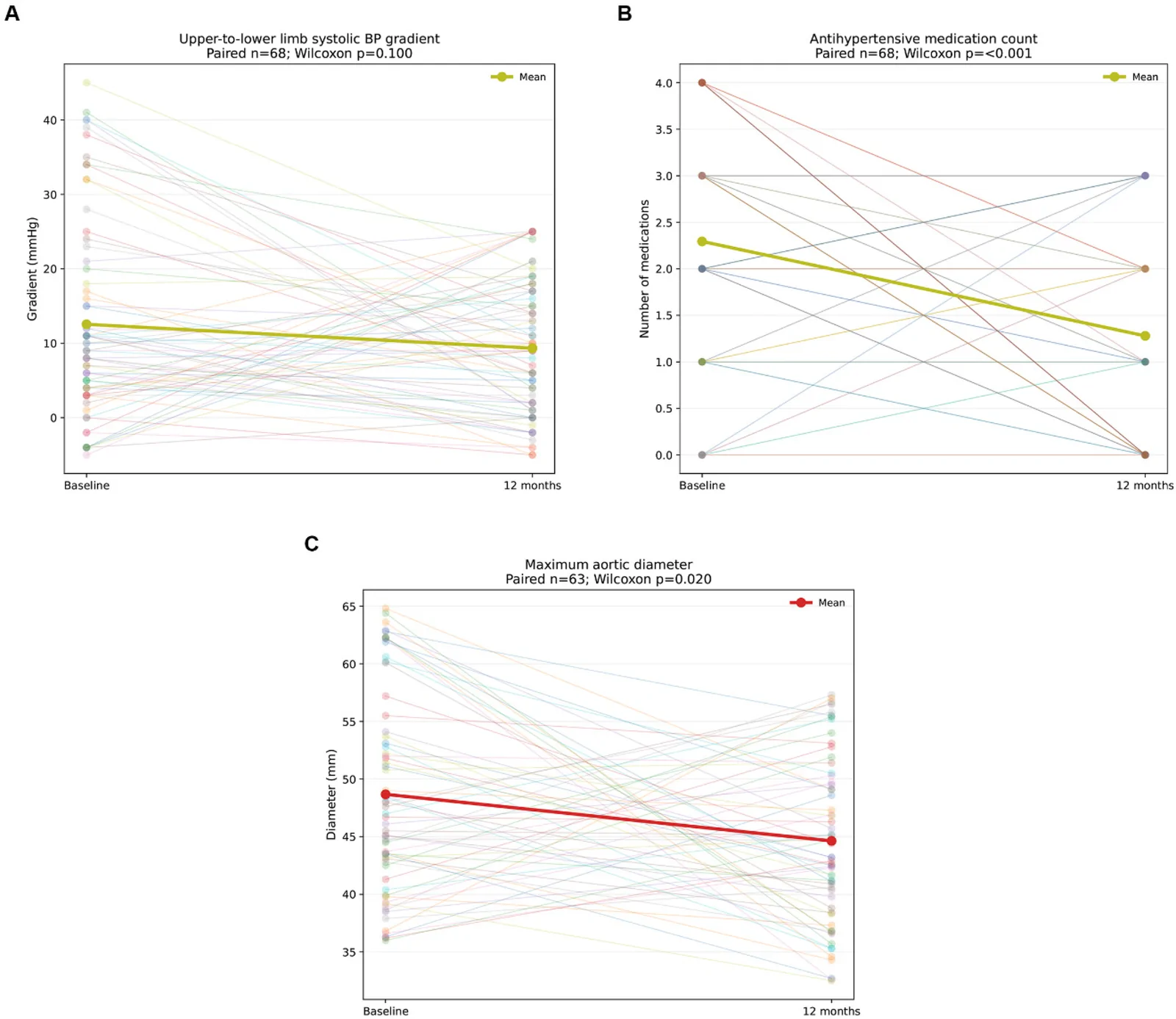
Paired changes in clinical and imaging outcomes from baseline to 12 months. Individual lines represent paired patient measurements, and the thick line indicates the mean. **(A)** Upper-to-lower limb systolic BP gradient (*n* = 68). **(B)** Antihypertensive medication count (*n* = 68). **(C)** Maximum aortic diameter (*n* = 63). *P* values were calculated using two-sided Wilcoxon signed-rank tests.

Paired echocardiographic data were available for 11 patients. Aortic velocity decreased from 2.2 ± 0.8 to 1.7 ± 0.5 m/s, but this change was not statistically significant (*P* = 0.308). Similarly, peak aortic gradient decreased from 21.9 ± 15.1 to 13.1 ± 7.2 mmHg without reaching statistical significance (*P* = 0.240). Clinical and hemodynamic outcomes are presented in Supplementary Table 3. Individual paired changes in the upper-to-lower-limb systolic blood-pressure gradient and antihypertensive medication count are illustrated in Figures 2A,B respectively.

### Aortic imaging outcomes

Aortic imaging within the strict 12-month window was available for 63 patients. Maximum aortic diameter decreased from 48.7 ± 8.7 mm at baseline to 44.6 ± 7.1 mm at 12 months, corresponding to a paired change of −4.0 ± 12.1 mm (*P* = 0.020). The individual paired measurements and mean change are shown in Figure 2C.

Among the 40 patients with paired true- and false-lumen measurements, mean true-lumen diameter increased from 22.8 ± 3.9 to 24.1 ± 4.0 mm, although the change did not reach statistical significance (*P* = 0.061). Mean false-lumen diameter decreased from 22.6 ± 6.8 to 15.8 ± 5.5 mm, with a paired change of −6.8 ± 9.6 mm (*P* < 0.001). Among 23 patients with paired aneurysm-sac measurements, mean sac diameter decreased from 53.7 ± 8.6 to 45.2 ± 6.8 mm, corresponding to a change of −8.5 ± 13.4 mm (*P* = 0.010).

False-lumen status changed significantly between baseline and 12 months among the 40 patients with paired classifications (*P* = 0.020). The proportion with a patent false lumen decreased from 32.5% to 10.0%, whereas the proportion with complete false-lumen thrombosis increased from 32.5% to 55.0%. The proportion with partial thrombosis remained 35.0%.

At 12 months, favourable aortic remodelling was observed in 37 of 63 patients (58.7%), stable remodelling in 22 (34.9%), and unfavourable remodelling in four (6.3%). Type I and type II endoleaks were identified in four (6.3%) and seven (11.1%) patients, respectively. No type III endoleaks were detected at the 12-month assessment. At the latest imaging assessment, favourable remodelling was present in 43 of 79 patients (54.4%), while type I, type II, and type III endoleaks were present in four (5.1%), nine (11.4%), and one (1.3%) patient, respectively. Main-graft migration was observed in three patients at both 12 months and latest imaging, and no main-graft fractures were identified. Detailed aortic imaging outcomes are presented in Supplementary Table 4.

### Target-vessel imaging outcomes

Target-vessel imaging within the prespecified 10–14-month assessment window was available for 63 patients and included 107 assessed target vessels. All 107 assessed target vessels were patent (100.0%; 95% CI, 96.5–100.0). Neither of the two patients who experienced target-vessel occlusion within 30 days had evaluable target-vessel CTA within this assessment window and therefore neither contributed to the 12-month patency denominator. At the patient level, all assessed target vessels were patent in each of the 63 patients (63/63, 100.0%; 95% CI, 94.3–100.0).

The latest target-vessel assessment included 79 patients and 134 branches. Of these, 132 branches remained patent, producing a per-vessel patency rate of 98.5% (95% CI 94.7–99.6). All assessed branches were patent in 77 of 79 patients, corresponding to a patient-level patency rate of 97.5% (95% CI 91.2–99.3).

Branch stenosis of at least 50% was present in five of 107 branches (4.7%) at 12 months and five of 134 branches (3.7%) at latest imaging. Branch endoleak was observed in four branches (3.7%) at 12 months and six (4.5%) at latest imaging. Branch kinking or compression was present in three branches (2.8%) at 12 months and eight (6.0%) at latest imaging. Target-vessel imaging outcomes are provided in Supplementary Table 5.

### Mortality and reintervention during follow-up

The median clinical follow-up duration was 19.0 months. Four patients died during follow-up, corresponding to an all-cause mortality rate of 4.9% (95% CI 1.9–12.0). One death was classified as aortic-related.

Seven patients (8.6%; 95% CI 4.2–16.8) underwent a first aortic- or target-vessel-related reintervention at a median of 10.3 months after the index procedure. Indications included type II endoleak in two patients, bridging-stent stenosis in one, persistent false-lumen perfusion in one, type I endoleak in one, bridging-stent migration with stenosis in one, and persistent type II endoleak with continued false-lumen perfusion in one. The two target-vessel occlusions recorded within 30 days were managed conservatively and did not require an additional endovascular or surgical procedure; therefore, they did not meet the prespecified definition of a qualifying reintervention and are not included among the indications for reintervention. Six reinterventions were performed endovascularly, whereas one patient required open conversion.

**Figure 3:**
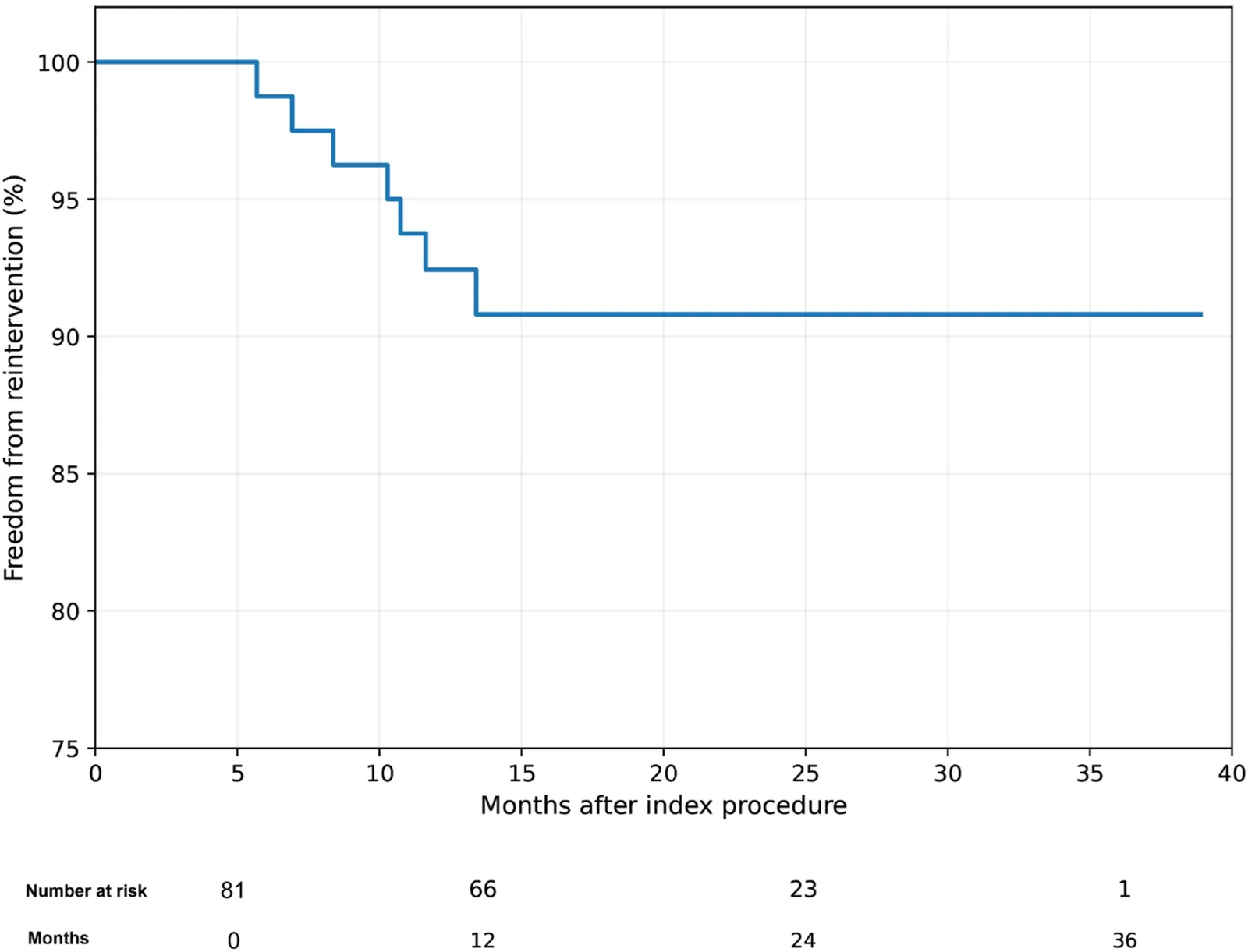
Freedom from first aortic- or branch-related reintervention. Kaplan–Meier estimates were 92.4% at 12 months and 90.8% at 24 months. Numbers at risk are shown below the curve; the 36-month estimate should be interpreted cautiously because only one patient remained at risk.

Kaplan–Meier freedom from first aortic- or branch-related reintervention was 92.4% at 12 months and 90.8% at 24 months. The corresponding numbers at risk were 66 and 23. A 36-month estimate was not reported because only one patient remained at risk at that time point. The Kaplan–Meier curve is shown in Figure 3.

**Figure 4:**
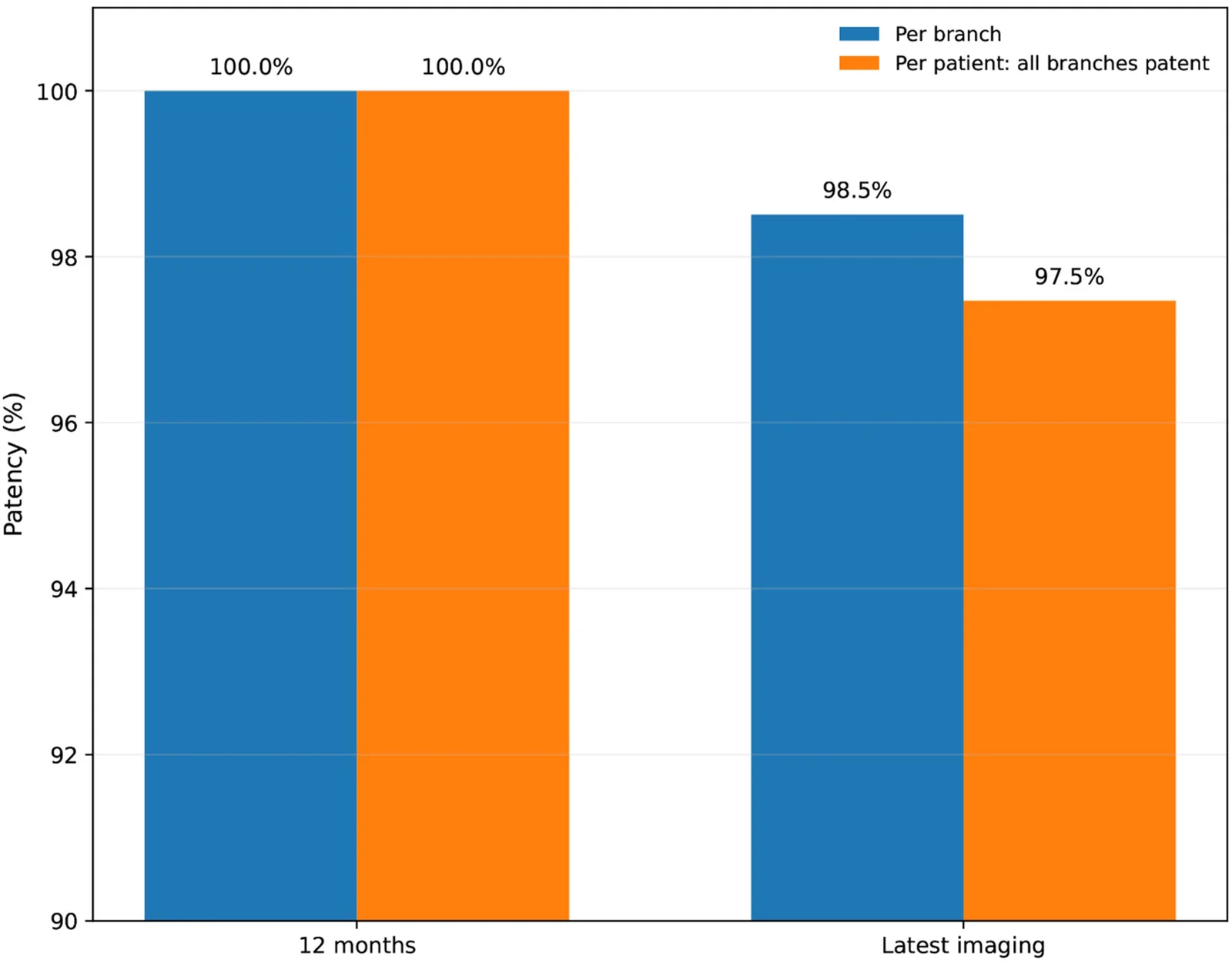
Target-vessel patency. At 12 months, patency was 100.0% per-vessel (107/107) and per patient (63/63). At latest imaging, patency was 98.5% per-vessel (132/134) and 97.5% per patient (77/79).

The per-vessel and patient-level target-vessel patency rates at 12 months and at the latest imaging assessment are summarised graphically in Figure 4.

### Exploratory subgroup analyses

Exploratory subgroup analyses according to underlying pathology, dissection chronicity, and fenestration technique are presented in Supplementary Table 6. No statistically significant differences were detected in procedure duration, technical success, or 30-day major adverse events across the three underlying pathology groups (*P* = 0.263, *P* = 0.261, and *P* = 0.149, respectively). Among the 53 patients with a dissection component, corresponding comparisons among acute, subacute, and chronic dissection were also not statistically significant (*P* = 0.691, *P* = 0.208, and *P* = 0.693, respectively).

Among the two principal fenestration techniques, laser and needle fenestration showed no statistically significant differences in procedure duration [median 205 [IQR 162–246] vs. 193 [129.5–235.3] minutes; *P* = 0.241], technical success [39/47 [83.0%] vs. 25/28 [89.3%]; *P* = 0.522], or 30-day major adverse events [10/47 [21.3%] vs. 7/28 [25.0%]; *P* = 0.779]. The six procedures classified as ‘other’ were reported descriptively because this category was small and included heterogeneous techniques.

## Discussion

### Principal findings

This study provides medium-term device-specific evidence on SilverFlow covered bridging stents used for *in-situ* fenestrated aortic arch repair. Procedural execution was successful in almost all patients, whereas the lower composite technical-success rate primarily reflected the strict inclusion of completion type I and III endoleaks. Target-vessel patency remained high among vessels with available follow-up imaging, while early occlusion and later structural abnormalities showed that patency alone does not fully characterize bridging-stent performance. Follow-up imaging also demonstrated changes consistent with aortic remodelling, although reinterventions and persistent device- or aorta-related abnormalities emphasize the need for continued surveillance. These findings extend the limited SilverFlow-specific literature beyond immediate procedural outcomes but should be interpreted in the context of the heterogeneous population and single-arm study design (11).

### Technical success and procedural feasibility

For context, previous studies have reported technical success rates of approximately 94% in pooled analyses and 97.3% in a 148-patient laser-fenestration series (7, 16). In the present cohort, technical success was 86.4% under the strict composite definition used in this study. Cross-study comparisons should be interpreted cautiously because of differences in patient selection, underlying pathology, fenestration technique, devices, and endpoint definitions. Deployment at the intended location, completion of all planned fenestrations, and immediate patency of all intended target vessels were each achieved in 98.8% of patients. However, any type I or type III completion endoleak, including a minor type Ia endoleak, resulted in classification as technical failure. Thus, the composite technical-success rate reflected both successful execution of the fenestration procedure and the immediate sealing performance of the complete reconstruction.

Completion endoleak was the principal factor reducing the composite technical-success rate. Published endoleak rates after *in-situ* fenestration are generally lower, although direct comparison is limited because some studies report only persistent or untreated endoleaks, whereas the present analysis recorded any endoleak detected on completion angiography (9, 16). Minor type Ia endoleaks may resolve during subsequent graft expansion and remodelling (17); nevertheless, their inclusion as technical failures provided a conservative assessment of immediate sealing performance.

The near-universal completion of the planned fenestrations is notable because more than half of the cohort underwent reconstruction of two or three supra-aortic vessels. Multivessel reconstruction increases procedural complexity because it requires repeated graft penetration, branch catheterisation, and accurate bridging-stent deployment while cerebral and upper-limb perfusion are being restored. Previous work has suggested that procedural complexity and complication rates may vary according to the underlying pathology and characteristics of the reconstruction (7). In the present exploratory analyses, procedure duration, technical success, and 30-day major adverse events did not differ significantly across pathology or dissection-chronicity groups, while laser and needle fenestration showed similar results for these endpoints. These findings are reassuring but should not be interpreted as evidence of equivalence, because the study was not powered to detect moderate subgroup differences.

### Early safety outcomes

No intraoperative, in-hospital, or 30-day deaths occurred in the present cohort, in which 46.9% of procedures were urgent or emergent and both dissection and aneurysmal disease were represented. Previous systematic reviews have reported pooled 30-day mortality of approximately 2%, whereas individual needle- and laser-fenestration series have reported rates of approximately 2.9%–3.5% (7, 9, 18). These published rates provide clinical context but are not directly comparable with the present cohort because of differences in case mix, procedural techniques, outcome definitions, and study design. In the absence of a concurrent comparator, the present findings cannot establish a mortality advantage over open, hybrid, or alternative total endovascular approaches.

he 30-day major adverse-event composite occurred in 25.9% of patients. A previous meta-analysis reported a lower pooled estimate (16); however, this estimate is not directly comparable with the present result because of differences in endpoint definitions, patient populations, and procedural characteristics. Access-site complications were evaluated and reported separately and were not included in the prespecified composite. Acute kidney injury was the most frequent component and may reflect the combined effects of contrast exposure, procedural duration, baseline renal vulnerability, and the physiological stress associated with complex arch intervention. These neurological events remain clinically important because arch instrumentation, temporary target vessel coverage, wire and catheter manipulation, embolization, and changes in cerebral or spinal collateral perfusion are intrinsic concerns during complex arch repair. The findings reinforce the importance of careful anatomical selection, procedural planning, minimization of target-vessel ischemia time, and performance by teams experienced in cerebral and spinal-cord risk management.

### Target-vessel patency and branch-related complications

Target-vessel patency was high among vessels with available follow-up imaging. All 107 target vessels assessed within the prespecified 12-month window were patent, and 132 of 134 assessed vessels remained patent at the latest imaging assessment. Importantly, these imaging-based estimates should be distinguished from the two target-vessel occlusions recorded as early 30-day safety events; neither of those patients had evaluable target-vessel CTA within the 12-month assessment window and therefore neither contributed to the 12-month denominator. These findings are broadly consistent with previously reported mid-term patency after *in-situ* fenestrated reconstruction (9, 17).

Patency alone, however, does not fully describe bridging-stent performance. Follow-up imaging identified target-vessel stenosis, bridging-stent-associated endoleak, and kinking or compression, with kinking or compression more frequently observed at the latest assessment than within the 12-month window. Potential contributors include changes in arch geometry and aortic remodelling, interaction between the main endograft and bridging stents, and pulsatile or respiratory motion. Because the timing of latest imaging varied among patients, this apparent increase cannot be interpreted as a formal longitudinal incidence. Nevertheless, the presence of structural abnormalities despite preserved flow supports continued imaging surveillance of both the reconstructed target vessel and bridging stent.

### Aortic remodelling

Paired imaging demonstrated changes consistent with reduced pressurisation of the treated aortic segment, including reductions in maximum aortic, false-lumen, and aneurysm-sac dimensions together with progression toward complete false-lumen thrombosis. The combination of false-lumen contraction, increased thrombosis, and reduction in overall aortic dimensions provides a more informative assessment of remodelling than any single measurement and is biologically consistent with exclusion of the proximal lesion while preserving supra-aortic perfusion.

Interpretation should nevertheless account for the heterogeneous underlying pathology. False-lumen dimensions and thrombosis status principally apply to patients with dissection, whereas aneurysm-sac measurements reflect a different remodelling process. Remodelling was also not uniformly favourable, and persistent or recurrent endoleak remained detectable during follow-up. These observations reinforce the need for serial imaging rather than implying complete or permanent exclusion of the underlying aortic disease.

### Clinical and haemodynamic outcomes

Upper- and lower-limb blood pressure and antihypertensive treatment burden decreased during follow-up, whereas the upper-to-lower-limb pressure gradient did not change significantly. These observations may reflect several factors, including recovery from the presenting aortic syndrome, changes in antihypertensive therapy, and altered aortic haemodynamics. Because antihypertensive management was neither randomised nor protocol-controlled and retrospective medication records could not fully capture dose changes or adherence, these findings should be considered exploratory and should not be interpreted as a direct treatment effect of the SilverFlow-based reconstruction.

### Reintervention and mid-term durability

Aortic- or target-vessel-related reinterventions during follow-up arose from both the aortic seal and target-vessel reconstruction, including endoleak, persistent false-lumen perfusion, bridging-stent stenosis, and migration. Most were managed endovascularly, although one patient required open conversion. Published reintervention rates after other fenestrated arch-repair strategies are of a similar order of magnitude, but differences in follow-up duration and endpoint definitions limit direct comparison (18, 19). Importantly, the apparent stability of the Kaplan–Meier curve after the early follow-up period should not be interpreted as evidence that late failure is unlikely because relatively few patients remained at risk beyond 24 months. Longer surveillance is required to assess late endoleak, bridging-stent deformation, migration, fracture or stenosis, and continued aortic enlargement.

### Clinical implications

Open total arch replacement remains the reference treatment for many patients with extensive aortic arch aneurysm or dissection, but its invasiveness and requirement for cardiopulmonary bypass and circulatory-arrest strategies may limit its suitability in older or high-risk patients. Total endovascular and hybrid approaches have consequently expanded, although the evidence supporting *in-situ* fenestration remains dominated by retrospective, single-centre studies with heterogeneous devices and outcome definitions (20, 21).

*In-situ* fenestration using readily available endografts and covered bridging stents may be particularly relevant when urgent aortic arch repair precludes manufacture of a patient-specific device. Its use should nevertheless remain concentrated in experienced multidisciplinary aortic centres because treatment requires precise graft penetration and target-vessel reconstruction together with the ability to manage neurological complications, endoleak, target-vessel failure, and potential open conversion. The present findings also indicate that procedural success should be assessed beyond completion of fenestration to include immediate sealing, early clinical safety, longitudinal target-vessel and bridging-stent morphology, aortic remodelling, and freedom from reintervention.

### Study limitations

This study has several limitations. Its retrospective, single-centre, single-arm design is susceptible to selection bias, information bias, and unmeasured confounding. Treatment selection was based on local expertise, anatomical suitability, and clinical judgement, and the cohort may not represent patients treated at centers with different experience or device availability. There was no concurrent open, hybrid, chimney, custom fenestrated/branched, or alternative endovascular comparison group; consequently, the observed procedural, clinical, and imaging outcomes cannot be interpreted as demonstrating superiority, non-inferiority, or comparative safety or effectiveness relative to other aortic arch repair strategies. Comparisons with previously published series were therefore used only to provide clinical context and should be interpreted cautiously because of differences in patient selection, pathology, procedural technique, devices, endpoint definitions, and follow-up duration.

The sample size was modest, and only seven first reintervention events occurred, precluding reliable multivariable modelling of predictors of treatment failure or reintervention. The population was heterogeneous with respect to pathology, dissection chronicity, procedural urgency, landing zone, number of reconstructed vessels, and fenestration method. Although exploratory subgroup analyses were performed according to underlying pathology, dissection chronicity, and fenestration technique, several subgroups were small and the number of outcome events was limited. In particular, only eight patients had dissection with aneurysmal degeneration and only six underwent techniques classified as ‘other.’ The absence of statistically significant subgroup differences should therefore not be interpreted as evidence of equivalence or as excluding clinically meaningful effects of pathology or technique. Residual confounding by anatomy, urgency, landing zone, and reconstruction complexity also remains possible.

Follow-up availability differed across outcomes. Strict 12-month clinical and imaging assessments were available for 68 and 63 patients, respectively, and paired analyses included only patients with complete measurements within the prespecified window. Patients without imaging may have differed systematically from those who returned, introducing attrition bias. Latest imaging was obtained at variable times, making it unsuitable for formal paired hypothesis testing. The high target-vessel patency estimates apply only to assessed branches and should not be assumed to represent unobserved branches.

Outcome ascertainment was based on retrospective clinical and imaging records. The absence of standardized prospective neurological assessment, medication titration, and independent external imaging adjudication may have introduced measurement variability. In addition, the strict technical-success definition differed from some previous reports, particularly through the inclusion of minor completion type Ia endoleaks as technical failures, which limits direct comparison.

The median follow-up of 19 months was sufficient to evaluate early and part of the mid-term experience but was inadequate to establish long-term durability. The small numbers at risk beyond 24 months make the later Kaplan–Meier estimates unstable. Finally, multiple secondary and exploratory outcomes were analyzed without adjustment for multiplicity, and statistically significant paired findings should therefore be interpreted alongside their clinical magnitude and the broader pattern of results.

## Conclusions

In this single-centre cohort, *in-situ* fenestration using SilverFlow covered bridging stents achieved successful deployment and immediate target-vessel patency in almost all patients, while target-vessel patency remained high among vessels with available follow-up imaging. Follow-up imaging demonstrated reductions in aortic, false-lumen, and aneurysm-sac dimensions together with an increase in complete false-lumen thrombosis. The composite technical success rate was 86.4% under the strict prespecified definition, and reinterventions and late aortic or target-vessel abnormalities occurred during follow-up. These findings support the feasibility of this strategy in experienced centres but do not establish comparative safety or effectiveness. Prospective multicentre studies with standardised definitions, appropriate comparator groups, independent imaging assessment, and longer follow-up are required.

## Data Availability

The raw data supporting the conclusions of this article will be made available by the authors, without undue reservation.
