# Supplementary material for: Medium-term clinical and imaging outcomes of SilverFlow covered bridging stent implantation using in situ fenestration for aortic arch lesions: a single-centre retrospective cohort study
Source: Front Cardiovasc Med. 2026 Aug 31;13:1945990. doi: 10.3389/fcvm.2026.1945990 (PMC13572141; doi:10.3389/fcvm.2026.1945990)
Supplement: Supplementary file 1 [file Datasheet1.docx]

| **Supplementary Table 1.** Procedural and device characteristics | | |
| --- | --- | --- |
| **Characteristic** | **Value** | **Available n** |
| **Procedure characteristics** | | |
| Procedure urgency: elective | 43/81 (53.1%) | 81 |
| Procedure urgency: urgent | 30/81 (37.0%) | 81 |
| Procedure urgency: emergency | 8/81 (9.9%) | 81 |
| Anaesthesia: general | 75/81 (92.6%) | 81 |
| Anaesthesia: sedation | 4/81 (4.9%) | 81 |
| Anaesthesia: local | 2/81 (2.5%) | 81 |
| Access: Percutaneous femoral | 55/81 (67.9%) | 81 |
| Access: Surgical femoral cutdown | 9/81 (11.1%) | 81 |
| Access: Iliac | 8/81 (9.9%) | 81 |
| Access: Brachial | 6/81 (7.4%) | 81 |
| Access: Axillary | 3/81 (3.7%) | 81 |
| **Lesion location and fenestration** | | |
| Primary lesion location: Zone 0-1 | 22/81 (27.2%) | 81 |
| Primary lesion location: Zone 1-2 | 22/81 (27.2%) | 81 |
| Primary lesion location: Zone 2 | 19/81 (23.5%) | 81 |
| Primary lesion location: Distal arch / proximal descending | 18/81 (22.2%) | 81 |
| Ishimaru zone 0 | 21/81 (25.9%) | 81 |
| Ishimaru zone 1 | 16/81 (19.8%) | 81 |
| Ishimaru zone 2 | 20/81 (24.7%) | 81 |
| Ishimaru zone 3 | 24/81 (29.6%) | 81 |
| Target vessels, n=1 | 38/81 (46.9%) | 81 |
| Target vessels, n=2 | 27/81 (33.3%) | 81 |
| Target vessels, n=3 | 16/81 (19.8%) | 81 |
| Fenestration method: laser | 47/81 (58.0%) | 81 |
| Fenestration method: needle | 28/81 (34.6%) | 81 |
| Fenestration method: other | 6/81 (7.4%) | 81 |
| **Main Aortic Endograft** | | |
| Main endograft | Ankura Thoracic Stent Graft System (LifeTech Scientific) | **81** |
| Main-endograft sizing strategy: dissection | Approximately 10% oversizing | **-** |
| Main-endograft sizing strategy: aneurysmal disease | Approximately 20% oversizing | **-** |
| Minimum intended proximal sealing length | Approximately 15 mm | **-** |
| **SilverFlow covered bridging stents** | | |
| Total SilverFlow covered branch stents | 140 | 81 |
| Branch stents per patient | 2 [1–2] | 81 |
| Target branch: Inominate | 50/140 (35.7%) | 140 |
| Target branch: LCCA | 50/140 (35.7%) | 140 |
| Target branch: LSA | 40/140 (28.6%) | 140 |
| Nominal branch-stent diameter, mm | 10.0 [8.0–12.0] | 140 |
| Nominal branch-stent length, mm | 50.0 [40.0–60.0] | 140 |
| **Intraoperative metrics** | | |
| Systemic heparin administered | 81/81 (100.0%) | 81 |
| Maximum activated clotting time, s | 342 [294–380] | 81 |
| Main-endograft post-dilatation performed | 67/81 (82.7%) | 81 |
| Final main-endograft dilation diameter, mm | 34.9 [32.2–36.9] | 67 |
| Final main-endograft dilation pressure, atm | 6.0 [5.0–9.0] | 67 |
| Residual pressure gradient, mmHg | 7.0 [3.0–12.0] | 81 |
| Procedure duration, min | 203 [154–245] | 81 |
| Fluoroscopy time, min | 68.2 [48.9–85.2] | 81 |
| Contrast volume, mL | 176 [120–227] | 81 |
| Estimated blood loss, mL | 335 [227–492] | 81 |
| **Technical outcomes** | | |
| Technical success | 70/81 (86.4%; 95% CI 77.3–92.2) | 81 |
| Stent deployed at intended location | 80/81 (98.8%) | 81 |
| All planned fenestrations completed | 80/81 (98.8%) | 81 |
| All target branches patent on completion | 80/81 (98.8%) | 81 |
| Completion endoleak: any | 12/81 (14.8%) | 81 |
| Completion endoleak: Type I | 1/81 (1.2%) | 81 |
| Completion endoleak: Type Ia (minor) | 7/81 (8.6%) | 81 |
| Completion endoleak: Type II | 1/81 (1.2%) | 81 |
| Completion endoleak: Type III | 3/81 (3.7%) | 81 |
| Intraoperative open conversion | 0/81 (0.0%) | 81 |
| Intraoperative death | 0/81 (0.0%) | 81 |
| *Footnotes: Continuous procedural metrics are median [IQR]; categorical values are n/N (%).* | | |

| **Supplementary Table 2.** Safety, mortality, and reintervention outcomes | | | | | | | | |  |
| --- | --- | --- | --- | --- | --- | --- | --- | --- | --- |
| **Outcome** | | | | **Value** | | | **Denominator / risk set** | |  |
| **Index admission** | | | | | | | | |  |
| Length of hospital stay, days | | | | 16 [11–22] | | | 81 | |  |
| ICU admission | | | | 55/81 (67.9%) | | | 81 | |  |
| ICU duration, days | | | | 1 [0–5] | | | 81 | |  |
| In-hospital death | | | | 0/81 (0.0%) | | | 81 | |  |
| 30-day death | | | | 0/81 (0.0%) | | | 81 | |  |
| 30-day readmission | | | | 11/81 (13.6%) | | | 81 | |  |
| **30-day safety outcomes** | | | | | | | | |  |
| Major adverse-event composite | | | | 21/81 (25.9%; 95% CI 17.6–36.4) | | | 81 | |  |
| Stroke or TIA | | | | 3/81 (3.7%) | | | 81 | |  |
| Spinal-cord ischaemia | | | | 2/81 (2.5%) | | | 81 | |  |
| Acute kidney injury | | | | 9/81 (11.1%) | | | 81 | |  |
| Myocardial infarction | | | | 0/81 (0.0%) | | | 81 | |  |
| Respiratory failure | | | | 4/81 (4.9%) | | | 81 | |  |
| Retrograde type A dissection | | | | 0/81 (0.0%) | | | 81 | |  |
| Aortic injury | | | | 2/81 (2.5%) | | | 81 | |  |
| Target-vessel occlusion | | | | 2/81 (2.5%) | | | 81 | |  |
| Unplanned reintervention | | | | 1/81 (1.2%) | | | 81 | |  |
| Open conversion | | | | 0/81 (0.0%) | | | 81 | |  |
| Access complication | | | | 6/81 (7.4%) | | | 81 | |  |
| Unplanned reintervention status unknown | | | | 3/81 (3.7%) | | | 81 | |  |
| **Complications during total follow-up** | | | | | | | | |  |
| Any complication, patients | | | | 24/81 (29.6%; 95% CI 20.8–40.3) | | | 81 | |  |
| Total complication events | | | | 28 | | | 81 | |  |
| Major complication, patients | | | | 16/81 (19.8%) | | | 81 | |  |
| Serious adverse event, patients | | | | 14/81 (17.3%) | | | 81 | |  |
| Device-related event, patients | | | | 6/81 (7.4%) | | | 81 | |  |
| Procedure-related event, patients | | | | 20/81 (24.7%) | | | 81 | |  |
| Event subtype: AKI stage 2-3 | | | | 7 events | | | 81 | |  |
| Event subtype: Respiratory failure | | | | 4 events | | | 81 | |  |
| Event subtype: Access site hematoma/pseudoaneurysm | | | | 4 events | | | 81 | |  |
| Event subtype: Type II endoleak | | | | 3 events | | | 81 | |  |
| Event subtype: Aortic injury | | | | 2 events | | | 81 | |  |
| Event subtype: Branch occlusion | | | | 2 events | | | 81 | |  |
| Event subtype: Graft migration | | | | 2 events | | | 81 | |  |
| Event subtype: Spinal cord ischaemia | | | | 2 events | | | 81 | |  |
| Event subtype: Stroke | | | | 2 events | | | 81 | |  |
| **Mortality and reintervention during follow-up** | | | | | | | | |  |
| Median follow-up, months | | | | 19.0 [13.4–25.7] | | | 81 | |  |
| All-cause death | | | | 4/81 (4.9%; 95% CI 1.9–12.0) | | | 81 | |  |
| Aortic-related death | | | | 1/81 (1.2%) | | | 81 | |  |
| Any aortic- or branch-related reintervention | | | | 7/81 (8.6%; 95% CI 4.2–16.8) | | | 81 | |  |
| Time to first reintervention, months | | | | 10.3 [7.7–11.2] | | | 7 | |  |
| Reintervention indication: Type II endoleak | | | | 2 events | | | 7 | |  |
| Reintervention indication: Branch stent stenosis | | | | 1 events | | | 7 | |  |
| Reintervention indication: Persistent false-lumen perfusion | | | | 1 events | | | 7 | |  |
| Reintervention indication: Type I endoleak | | | | 1 events | | | 7 | |  |
| Reintervention indication: Branch stent migration with stenosis | | | | 1 events | | | 7 | |  |
| Reintervention indication: Persistent type II endoleak | | | | 1 events | | | 7 | |  |
| Freedom from reintervention at 12 months | | | | 92.4% (95% CI 83.9–96.5) | | | at risk=66 | |  |
| Freedom from reintervention at 24 months | | | | 90.8% (95% CI 81.6–95.5) | | | at risk=23 | |  |
| Freedom from reintervention at 36 months | | | | Not reported; only 1 patient remained at risk | | | at risk=1 | |  |
| *Footnote: The 30-day composite follows the endpoint definition. Event subtype rows report event counts. Both target-vessel occlusions were recorded within 30 days and were managed conservatively because collateral perfusion was adequate and no clinically significant cerebral or upper-limb ischaemia developed. Neither event required an additional endovascular or surgical procedure. Complication severity was classified according to the CIRSE Classification System, with CIRSE Grade ≥3 events classified as major complications. Serious adverse events were defined according to ISO 14155 criteria. Device-related and procedure-related events were those considered probably or definitely attributable to the implanted device or index procedure, respectively. Severity, seriousness, device relatedness, and procedure relatedness were assessed separately and were not mutually exclusive. Potential complication events were reviewed by two study investigators, with disagreements resolved by consensus. The prespecified 30-day major adverse-event composite comprised all-cause death, stroke or transient ischaemic attack, spinal-cord ischaemia, acute kidney injury, myocardial infarction, respiratory failure, retrograde type A dissection, major aortic injury, target-vessel occlusion, open conversion, or unplanned reintervention. Access-site complications were reported separately and were not included in the composite.* | | | | | | | | |  |
| **Supplementary Table 3. Follow up and Clinical-hemodynamic outcomes** | | | | | | | | | |
| **Outcome** | **Baseline** | **12 months** | **Latest** | | **Paired change at 12 months** | **P value** | | **Statistical method / notes** | |
| **Follow-up availability** | | | | | | | | | |
| Clinical assessment available | 81/81 baseline | 68/81 (84.0%) | 81/81 (100.0%) | | Not applicable | Not applicable | | Descriptive availability; strict 12-month window was 10–14 months. | |
| Aortic imaging available | 81/81 baseline | 63/81 (77.8%) | 79/81 (97.5%) | | Not applicable | Not applicable | | Descriptive availability; latest post-procedure imaging on or before 2026-04-15. | |
| Time from procedure to assessment, months | — | 11.7 [10.9–12.3] | 12.3 [11.2–21.2] | | Not applicable | Not applicable | | Descriptive timing only; no hypothesis test. | |
| **Clinical and hemodynamic outcomes** | | | | |  |  | |  | |
| Upper-limb systolic BP, mmHg | 159.0 ± 17.3 | 137.6 ± 13.4 | 138.3 ± 13.9 | | -21.4 ± 21.0 | <0.001 | | Two-sided Wilcoxon signed-rank test for baseline versus strict 12-month paired data. Paired n=68 at 12 months; n=81 at latest | |
| Lower-limb systolic BP, mmHg | 146.4 ± 22.9 | 128.3 ± 16.5 | 129.5 ± 16.3 | | -18.1 ± 26.3 | <0.001 | | Two-sided Wilcoxon signed-rank test for baseline versus strict 12-month paired data. Paired n=68 at 12 months; n=81 at latest | |
| Upper-to-lower limb systolic BP gradient, mmHg | 12.5 ± 13.2 | 9.3 ± 9.0 | 8.7 ± 9.1 | | -3.2 ± 16.1 | 0.1 | | Two-sided Wilcoxon signed-rank test for baseline versus strict 12-month paired data. Paired n=68 at 12 months; n=81 at latest | |
| Antihypertensive medications, n | 2.3 ± 1.3 | 1.3 ± 1.2 | 1.5 ± 1.2 | | -1.0 ± 1.9 | <0.001 | | Two-sided Wilcoxon signed-rank test for baseline versus strict 12-month paired data. Paired n=68 at 12 months; n=81 at latest | |
| Aortic velocity, m/s | 2.2 ± 0.8 | 1.7 ± 0.5 | 1.9 ± 0.6 | | -0.5 ± 1.0 | 0.308 | | Two-sided Wilcoxon signed-rank test for baseline versus strict 12-month paired data. Paired n=11 at 12 months; n=15 at latest | |
| Peak aortic gradient, mmHg | 21.9 ± 15.1 | 13.1 ± 7.2 | 15.6 ± 9.2 | | -8.9 ± 17.1 | 0.24 | | Two-sided Wilcoxon signed-rank test for baseline versus strict 12-month paired data. Paired n=11 at 12 months; n=15 at latest | |
| Hypertension present | 55/68 (80.9%) | 29/68 (42.6%) | 35/81 (43.2%) | | -26 patients; -38.2 percentage points | <0.001 | | Exact McNemar test in 68 paired patients; Yes→No: 33, No→Yes: 7. Latest value is descriptive. | |
| Antihypertensive therapy | 60/68 (88.2%) | 43/68 (63.2%) | 56/81 (69.1%) | | -17 patients; -25.0 percentage points | 0.002 | | Exact McNemar test in 68 paired patients; Yes→No: 23, No→Yes: 6. Latest value is descriptive. | |
| *Footnotes: P values compare baseline with the strict 12-month assessment only. The strict 12-month window was 10–14 months after the index procedure.; Continuous paired outcomes were tested with two-sided Wilcoxon signed-rank tests; paired binary outcomes used exact McNemar tests; false-lumen status used a global Stuart–Maxwell test.; Latest follow-up values are descriptive because the timing and available-case denominators vary among patients.; Not applicable indicates a descriptive availability measure, a post-implantation follow-up-only outcome, or an outcome without a valid pre-procedure comparator.* | | | | | | | | | |

|  | **Supplementary Table 4. Aortic imaging outcomes** | | | | | | | | | | | | | |
| --- | --- | --- | --- | --- | --- | --- | --- | --- | --- | --- | --- | --- | --- | --- |
|  | **Outcome** | **Baseline** | | **12 months** | | **Latest** | | **Paired change at 12 months** | | **P value** | | **Statistical method / notes** | | |
|  | **Aortic imaging outcomes** | | | | | | | | | | | | | |
|  | Maximum aortic diameter, mm | 48.7 ± 8.7 | | 44.6 ± 7.1 | | 43.7 ± 7.4 | | -4.0 ± 12.1 | | 0.02 | | Two-sided Wilcoxon signed-rank test for baseline versus strict 12-month paired data. Paired n=63 at 12 months; n=79 at latest | | |
|  | True-lumen diameter, mm | 22.8 ± 3.9 | | 24.1 ± 4.0 | | 25.0 ± 4.1 | | 1.3 ± 4.2 | | 0.061 | | Two-sided Wilcoxon signed-rank test for baseline versus strict 12-month paired data. Paired n=40 at 12 months; n=51 at latest | | |
|  | False-lumen diameter, mm | 22.6 ± 6.8 | | 15.8 ± 5.5 | | 15.2 ± 5.7 | | -6.8 ± 9.6 | | <0.001 | | Two-sided Wilcoxon signed-rank test for baseline versus strict 12-month paired data. Paired n=40 at 12 months; n=51 at latest | | |
|  | Aneurysm-sac diameter, mm | 53.7 ± 8.6 | | 45.2 ± 6.8 | | 46.6 ± 6.8 | | -8.5 ± 13.4 | | 0.01 | | Two-sided Wilcoxon signed-rank test for baseline versus strict 12-month paired data. Paired n=23 at 12 months; n=28 at latest | | |
|  | False-lumen status, overall paired distribution | 40 paired patients | | 40 paired patients | | 51 evaluable patients | | Global change in the 3-category distribution | | 0.02 | | Stuart–Maxwell test of marginal homogeneity for baseline versus strict 12 months. Latest distribution is descriptive. | | |
|  | False-lumen status: patent | 13/40 (32.5%) | | 4/40 (10.0%) | | 9/51 (17.6%) | | -9 patients; -22.5 percentage points | | Not separately tested | | Category-specific paired counts; use the global Stuart–Maxwell P value above. | | |
|  | False-lumen status: partially thrombosed | 14/40 (35.0%) | | 14/40 (35.0%) | | 15/51 (29.4%) | | 0 patients; 0.0 percentage points | | Not separately tested | | Category-specific paired counts; use the global Stuart–Maxwell P value above. | | |
|  | False-lumen status: completely thrombosed | 13/40 (32.5%) | | 22/40 (55.0%) | | 27/51 (52.9%) | | +9 patients; +22.5 percentage points | | Not separately tested | | Category-specific paired counts; use the global Stuart–Maxwell P value above. | | |
|  | Aortic remodelling: favourable | Not applicable | | 37/63 (58.7%) | | 43/79 (54.4%) | | Not applicable | | Not applicable | | Follow-up-only post-implantation outcome; no pre-procedure comparator. | | |
|  | Aortic remodelling: stable | Not applicable | | 22/63 (34.9%) | | 31/79 (39.2%) | | Not applicable | | Not applicable | | Follow-up-only post-implantation outcome; no pre-procedure comparator. | | |
|  | Aortic remodelling: unfavourable | Not applicable | | 4/63 (6.3%) | | 5/79 (6.3%) | | Not applicable | | Not applicable | | Follow-up-only post-implantation outcome; no pre-procedure comparator. | | |
|  | Type I endoleak | Not applicable | | 4/63 (6.3%) | | 4/79 (5.1%) | | Not applicable | | Not applicable | | Follow-up-only post-implantation outcome; no pre-procedure comparator. | | |
|  | Type II endoleak | Not applicable | | 7/63 (11.1%) | | 9/79 (11.4%) | | Not applicable | | Not applicable | | Follow-up-only post-implantation outcome; no pre-procedure comparator. | | |
|  | Type III endoleak | Not applicable | | 0/63 (0.0%) | | 1/79 (1.3%) | | Not applicable | | Not applicable | | Follow-up-only post-implantation outcome; no pre-procedure comparator. | | |
|  | Other endoleak | Not applicable | | 0/63 (0.0%) | | 0/79 (0.0%) | | Not applicable | | Not applicable | | Follow-up-only post-implantation outcome; no pre-procedure comparator. | | |
|  | Main-graft migration | Not applicable | | 3/63 (4.8%) | | 3/79 (3.8%) | | Not applicable | | Not applicable | | Follow-up-only post-implantation outcome; no pre-procedure comparator. | | |
|  | Main-graft fracture | Not applicable | | 0/63 (0.0%) | | 0/79 (0.0%) | | Not applicable | | Not applicable | | Follow-up-only post-implantation outcome; no pre-procedure comparator. | | |
|  |  | | | | | | | | | | | | | |
| **Supplementary Table 5.** Target-branch imaging outcomes | | | | | | | | | | | | | |  |
| **Outcome** | | | **Baseline** | | **12 months** | | **Latest** | | **Paired change at 12 months** | | **P value** | | **Statistical method / notes** |  |
| **Target-branch imaging outcomes** | | | | | | | | | | | | | |  |
| Patients with branch imaging | | | Not applicable | | 63 | | 79 | | Not applicable | | Not applicable | | Post-implantation branch-imaging outcome; descriptive reporting only. |  |
| Assessed branches | | | Not applicable | | 107 | | 134 | | Not applicable | | Not applicable | | Post-implantation branch-imaging outcome; descriptive reporting only. |  |
| Branch patency, per branch | | | Completion: not separately adjudicated per branch | | 107/107 (100.0%; 95% CI 96.5–100.0) | | 132/134 (98.5%; 95% CI 94.7–99.6) | | Not applicable | | Not applicable | | Per assessed branch; no separately adjudicated pre-implantation branch-stent baseline. |  |
| All assessed branches patent, per patient | | | 80/81 (98.8%; 95% CI 93.3–99.8) | | 63/63 (100.0%; 95% CI 94.3–100.0) | | 77/79 (97.5%; 95% CI 91.2–99.3) | | Not applicable | | Not applicable | | Completion angiography and follow-up CTA are different assessment timepoints/modalities; descriptive comparison only. |  |
| Branch stenosis ≥50%, per branch | | | Not applicable | | 5/107 (4.7%) | | 5/134 (3.7%) | | Not applicable | | Not applicable | | Post-implantation branch-imaging outcome; descriptive reporting only. |  |
| Branch endoleak, per branch | | | Not applicable | | 4/107 (3.7%) | | 6/134 (4.5%) | | Not applicable | | Not applicable | | Post-implantation branch-imaging outcome; descriptive reporting only. |  |
| Branch kinking/compression, per branch | | | Not applicable | | 3/107 (2.8%) | | 8/134 (6.0%) | | Not applicable | | Not applicable | | Post-implantation branch-imaging outcome; descriptive reporting only. |  |

*Footnotes: Patency rates are calculated among target vessels with evaluable imaging at the specified assessment. The two patients with target-vessel occlusion recorded within 30 days did not have evaluable target-vessel CTA within the prespecified 10–14-month window and therefore did not contribute to the 12-month patency denominator. Accordingly, the 12-month estimate represents patency among assessed target vessels rather than cumulative freedom from target-vessel occlusion.*

| **Supplementary Table 6.** Exploratory subgroup analyses according to underlying pathology, dissection chronicity, and fenestration technique | | | | |
| --- | --- | --- | --- | --- |
| **Subgroup** | **n** | **Procedure duration, min** | **Technical success** | **30-day major adverse events** |
| **Underlying pathology** | | | | |
| Aortic arch dissection | 45 | 199 [157–245] | 41/45 (91.1%) | 8/45 (17.8%) |
| Aortic arch aneurysm | 28 | 205 [153.5–237.8] | 22/28 (78.6%) | 10/28 (35.7%) |
| Dissection with aneurysmal degeneration | 8 | 252 [188–272.5] | 7/8 (87.5%) | 3/8 (37.5%) |
| P value | — | 0.263 | 0.261 | 0.149 |
| **Dissection chronicity*** | | | | |
| Acute | 15 | 199 [154.5–233.5] | 15/15 (100.0%) | 2/15 (13.3%) |
| Subacute | 16 | 184 [127.8–263.8] | 15/16 (93.8%) | 3/16 (18.8%) |
| Chronic | 22 | 206 [173–250.8] | 18/22 (81.8%) | 6/22 (27.3%) |
| P value | — | 0.691 | 0.208 | 0.693 |
| **Fenestration technique†** | | | | |
| Laser | 47 | 205 [162–246] | 39/47 (83.0%) | 10/47 (21.3%) |
| Needle | 28 | 193 [129.5–235.3] | 25/28 (89.3%) | 7/28 (25.0%) |
| Other | 6 | 243 [214.3–270.3] | 6/6 (100.0%) | 4/6 (66.7%) |
| P value‡ | — | 0.241 | 0.522 | 0.779 |
| *Footnotes: *Values are median [IQR] or n/N (%). Dissection chronicity was evaluated among the 53 patients with a dissection component, including patients with dissection and aneurysmal degeneration. †The “other” category comprised six patients treated using heterogeneous non-laser/non-needle fenestration techniques and is presented descriptively. ‡P values for fenestration technique compare laser with needle fenestration only. Procedure duration was compared using the Kruskal–Wallis test for the three-category pathology and chronicity analyses and the Mann–Whitney U test for laser versus needle fenestration. Categorical outcomes were compared using Fisher–Freeman–Halton exact tests for three-category comparisons and Fisher’s exact test for laser versus needle comparisons. All subgroup analyses were exploratory, and no multiplicity adjustment was applied.* | | | | |
